# Timing of intubation of pediatric hematopoietic cell transplant patients: an international survey

**DOI:** 10.3389/fonc.2024.1400635

**Published:** 2024-04-29

**Authors:** Janet Hume, Lexie Goertzen, Yvonne Avent, Marie E. Steiner, Jennifer McArthur

**Affiliations:** ^1^ Department of Pediatrics, Division of Critical Care Medicine, University of Minnesota/Masonic Children’s Hospital, Minneapolis, MN, United States; ^2^ Department of Pediatrics, Division of Critical Care and Pulmonary Medicine, St Jude Children’s Research Hospital, Memphis, TN, United States; ^3^ Department of Pediatrics, Division of Hematology/Oncology, University of Minnesota/Masonic Children’s Hospital, Minneapolis, MN, United States; ^4^ Department of Pediatrics, Division of Critical Care Medicine, University of Tennessee Health Sciences Center, Memphis, TN, United States

**Keywords:** hematopoietic cell transplant, intubation, mechanical ventilation, palliative care, non-invasive ventilation associated lung injury, oxygen toxicity

## Abstract

**Introduction:**

Retrospective data suggest that pediatric hematopoietic cell transplant (HCT) patients placed on non-invasive ventilation (NIV) prior to intubation have increased risk of mortality compared to patients who are intubated earlier in their course. The HCT-CI subgroup of the PALISI Network set out to gain a better understanding of factors that influence clinician’s decisions surrounding timing of intubation of pediatric HCT patients.

**Methods:**

We validated and distributed a brief survey exploring potential factors that may influence clinician’s decisions around timing of intubation of pediatric HCT patients with acute lung injury (ALI).

**Results:**

One hundred and four of the 869 PALISI Network’s members responded to the survey; 97 of these respondents acknowledged caring for HCT patients and were offered the remainder of the survey. The majority of respondents were PICU physicians (96%), with a small number of Advanced Practice Providers and HCT physicians. As expected, poor prognosis categories were perceived as a factors that delay timing to intubation whereas need for invasive procedures was perceived as a factor shortening timing to intubation. Concerns for oxygen toxicity or NIV-associated lung injury were not believed to influence timing of intubation.

**Discussion:**

Our survey indicates increased risk of ALI from prolonged NIV and oxygen toxicity in HCT patients are not a concern for most clinicians. Further education of pediatric ICU clinicians around these risk factors could lead to improvement in outcomes and demands further study. Additionally, clinicians identified concerns for the patient’s poor prognosis as a common reason for delayed intubation.

## Introduction

Delayed intubation and prolonged use of NIV and supplemental oxygen have been implicated as potential risk factors for poor outcome in pediatric hematopoietic cell transplant patients with Pediatric Acute Respiratory Distress Syndrome (PARDS) (1–3). Additionally, recent data has shown that lung injury from NIV may be underappreciated and contribute to the suboptimal outcomes for HCT patients who develop PARDS (4, 5). While much discussion has centered on the need for earlier intervention in this high-risk population (6), retrospective data suggests pediatric intensivists may intubate this population late in their PARDS course as evidenced by the very high rate of cardiac arrest during intubation when compared to the general pediatric population (1, 2, 7, 8). The Hematopoietic Cell Transplant and Cancer Immunotherapy (HCT-CI) subgroup of the Pediatric Acute Lung Injury and Sepsis Investigator’s (PALISI) Network performed a survey of pediatric intensivists and pediatric HCT clinicians within the PALISI Network to gain a better understanding of clinician’s beliefs surrounding the timing of intubation for pediatric HCT patients with PARDS. The purpose of the survey is to better inform future educational efforts as well as guide research efforts of the group aimed at improving outcomes for pediatric HCT patients with PARDS.

## Methods

Survey questions were written by members of the PALISI Network’s HCT-CI subgroup and validated through the following process: 1) potential questions drafted by working group to address possible factors influencing decision making around timing of intuabtion; 2) questions sent to 8 members of the HCT-CI subgroup’s Executive Committee for comments and revision; 3) questions asked to the University of Minnesota and St Jude Childrens Research Hospitals’ critical care teams including 10 physicians, 7 Advanced Practice Providers, one research coordinator, and 3 fellows resulting in 3 revisions to achieve uniformity in question interpretation. The final survey contained 19 questions which included 3 demographic questions regarding the respondent, 1 question addressing the respondent’s self-assessment of their own timing of intubation of HCT patients, 2 case scenarios developed to test whether engraftment status influenced decision making, and thirteen 5-point Likert scale questions investigating the factors the HCT-CI subgroup identified as potential influencers of decision making around timing of intubation. The survey was approved by the PALISI Network’s scientific review committee for distribution to its members. A cross sectional survey was undertaken with distribution to all members of the general PALISI Network, including the HCT-CI subgroup, through email with a link to RedCAP. Prior to distribution the survey was approved by the University of Minnesota IRB. Survey responses were anonymous and data were presented collectively through RedCAP. Statistical analysis is descriptive.

## Results

A total of 869 surveys were sent via email to members of the PALISI Network. One hundred and four members from 33 centers in 4 countries and 3 continents responded for a response rate of 12%. Of the 104 respondents, 97 cared for HCT patients and were then given the remainder of questions through branching logic. Of these 97 respondents, 82 were PICU attending physicians, 9 PICU fellows, 1 PICU advanced practice provider (APP), 3 HCT attending physicians, 1 HCT APP and 1 “other”.

**Figure 1:**
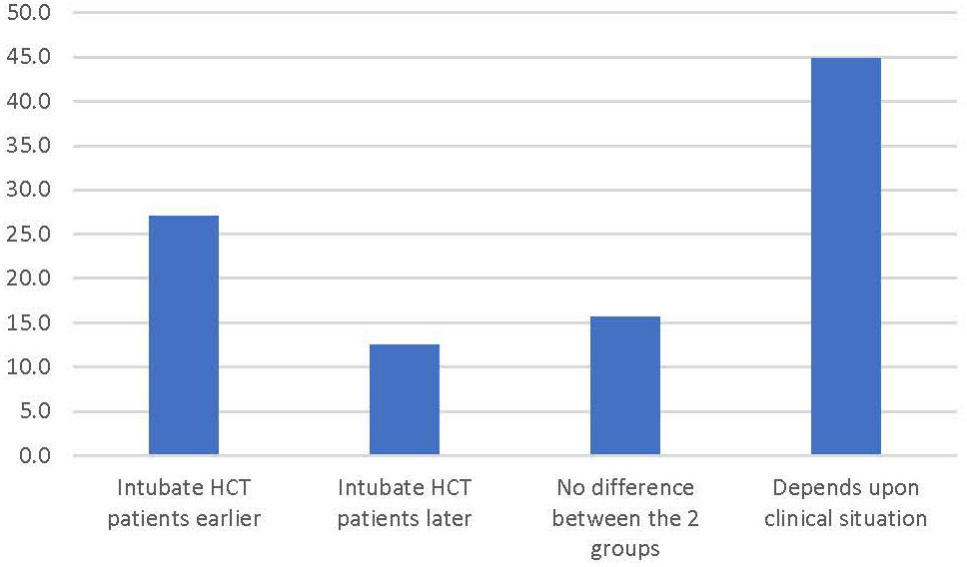
Percentage of respondents answering the survey prompt “In general, when comparing the timing of intubation for children who have respiratory failure after an HCT to the general pediatric population, I tend to…”.

After determining if respondents cared for HCT patients, they were asked to report their assessment of their own timing of intubation of HCT patients. The most popular answer was “depends on the situation” (44.8% of respondents) while 27.1% answered they intubate HCT patients earlier and 12.5% answered they intubated HCT patients later than the general PICU population (**Figure 1**).

The survey then presented the following clinical scenarios:

Scenario 1: Patient is a 6-year-old male who is Day +28 after allogeneic HCT. He is admitted to the PICU with respiratory distress. His current VS are T 37.2 P 140 R 50 BP 100/60 Oxygen Saturation 86%. He has been on the current BiPap settings (IPAP 18/EPAP 10 FiO2 0.7) for 6 hours. VBG shows pH 7.32/PCO2 58/HCO3 32. He is engrafted. What would most likely be your next plan?

Scenario 2: Patient is a 6-year-old male who is Day +40 after allogeneic HCT. He is admitted to the PICU with respiratory distress. His current VS are T 37.2 P 140 R 50 BP 100/60 Oxygen Saturation 86%. He has been on the current BiPap settings (IPAP 18/EPAP 10 FiO2 0.7) for 6 hours. VBG shows pH 7.32/PCO2 58/HCO3 32. He is not engrafted and there is concern he may have relapsed. What would most likely be your next plan?

**Figure 2:**
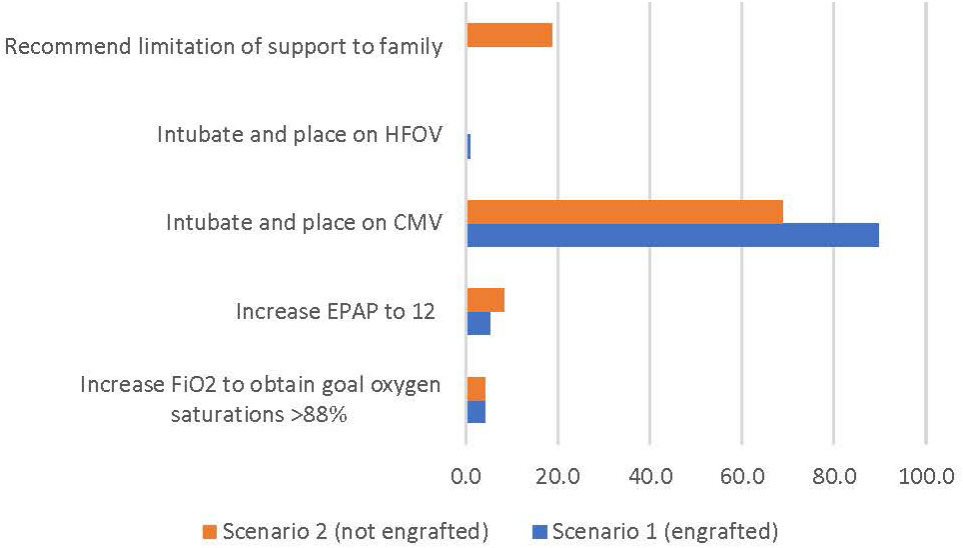
Percentage of respondents giving the survey response for clinical scenarios 1 and 2 (see text for description).

In the first scenario, 86 (89.6%) respondents indicated they would intubate the patient and place on conventional mechanical ventilation (CMV) with no respondents recommending limitation of support. In the second scenario where relapse was a consideration, 66 respondents (68.8%) elected to intubate and place on CMV with 18 (18.8%) recommending limitation of support (**Figure 2**).

We then asked questions regarding the role that different clinical factors played in determining timing of intubation (**Table 1**). Answers were on a 5-point Likert scale. The 2 factors that respondents most commonly responded “definitely plays a role” or “somewhat played a role” in delaying intubation were 1) patient with a poor prognosis that they felt should have limitations of support, but they hadn’t had time for discussion (89.78); and 2) patients they felt would not be able to extubated (93.8). While the 2 most common factors respondents answered “definitely” or “somewhat played a role” in intubating HCT patients earlier were 1) need for CRRT (63.5%) and 2) need for bronchoscopy (79.2%).

**Table 1:** Effect of specific clinical factors on timing of intubation in HCT patients.

	Definitely plays a role in delaying intubation	Slightly plays a role in delaying intubation	No impact	Slight role in decision to intubate earlier	Definite role in decision to intubate earlier
Medical team (PICU and/or HCT) feels patient should be DNR/DNI but there has not yet been a discussion of code status with patient/family	50 (51.5%)	37 (38.1%)	10 (10.3%)	0 (0%)	0 (0%)
Medical team (PICU and/or HCT) does not feel patient will be extubated once intubated, worried about loss of communication for patient with family	56 (57.7%)	35 (36.1%)	6 (6.2%)	0 (0%)	0 (0%)
Disagreement between family members regarding code status	17 (17.5%)	51 (52.6%)	29 (29.9%)	0 (0%)	0 (0%)
Disagreement between medical providers regarding appropriateness of invasive mechanical ventilation	18 (18.8%)	58 (60.4%)	16 (16.7%)	3 (3.1%)	1 (1%)
Concern for difficult airway and/or cardiac arrest with intubation procedure - waiting for additional help for procedure	25 (26%)	35 (36.5%)	18 (18.8%)	12 (12.5%)	6 (6.3%)
Concern for infection from invasive endotracheal tube/ventilator associated pneumonia	1 (1%)	13 (13.5%)	77 (80.2%)	3 (3.1%)	2 (2.1%)
Patient has not engrafted and engraftment unlikely	17 (17.7%)	36 (37.5%)	40 (41.7%)	2 (2.1%)	1 (1%)
Patient may have relapsed/have persistent primary disease for which HCT performed	23 (24%)	30 (31.3%)	39 (40.6%)	4 (4.2%)	0 (0%)
Concern for increased risk of oxygen toxicity in HCT patients	0 (0%)	2 (2.1%)	73 (76%)	18 (18.8%)	3 (3.1%)
Concern for cardiac arrest during intubation of HCT patients - better chance of survival without intubation & use NIV instead	7 (7.3%)	24 (25%)	45 (46.9%)	14 (14.6%)	6 (6.3%)
Concern for increased risk of lung injury with non-invasive ventilation in HCT patients	0 (0%)	7 (7.3%)	53 (55.2%)	28 (29.2%)	8 (8.3%)
Need for bronchoscopy to obtain diagnosis	0 (0%)	0 (0%)	20 (20.8%)	44 (45.8%)	32 (33.3%)
Need for CRRT to optimize fluid balance	0 (0%)	1 (1%)	34 (35.4%)	40 (41.7)	21 (21.9%)

The 2 factors which the PALISI HCT-CI subgroup have identified in previous retrospective studies to increase risk for poor outcomes in HCT patients requiring mechanical ventilation, prolonged oxygen exposure and NIV exposure prior to intubation, were not commonly identified as influencing behavior. Most respondents (76%) stated concern for increased risk of oxygen toxicity in HCT patients had no impact of their decision regarding timing of intubation with only 3.1% stating it “definitely plays a role” in the decision to intubate earlier. Additionally, 55.2% of respondents stated risk of lung injury from NIV had no impact on their decisions surrounding timing of intubation with only 8.3% stating it “definitely played a role” in their decision to intubate HCT patients earlier. Increased risk of peri-intubation cardiac arrest in the HCT population, also did not seem to play a significant role in decisions surrounding timing of intubation with nearly half (46.9%saying it had no impact and only 6.3% stating it “definitely played a role” in deciding to intubate earlier (**Table 1**).

## Discussion

It is well known that HCT patients requiring mechanical ventilation are at high risk of poorer outcomes than general medical PICU patients requiring mechanical ventilation (2, 3, 7). The PALISI Network’s HCT-CI subgroup has been committed to improving these outcomes since its inception in 2005. We and others have shown that HCT patients exposed to high levels of oxygen support or NIV prior to intubation are associated with worse outcomes (1, 2, 7, 9, 10). It is unclear if this is a causative relationship. However, given the high level of inflammatory response and oxidative stress that plague the HCT population, it is feasible these patients could be more susceptible to oxygen toxicity and NIV-induced lung injury than the general population. These factors do not seem to play a role in determining an earlier timing of intubation according to our survey results. Therefore, these are areas which may be important for our group and others to focus resources for research and education on in order to improve outcomes in this vulnerable population.

The factors most associated with delaying intubation center around physician concerns for a poor patient prognosis – that patients should have limitations of support or that they will be unable to extubate them. While this is understandable, it can be a self-fulfilling prophecy, preventing us from advancing the field. If we believe these patients can’t be saved, we don’t provide them with aggressive care, and they do poorly as we expect reinforcing our belief that they cannot be saved.

Limitations of the survey are the small number of respondents as well as few responses from HCT physicians. The small number of total respondents may be related to the requirement that respondents personally provide care to HCT patients, which is an unknown number, but likely a minority of PALISI members. HCT physicians also are an even smaller minority of PALISI members. However, the decisions around timing of intubation are mostly made by ICU physicians so lack of HCT clinician response likely does not have a large impact on the results. Additionally, actual clinician practice is often not concordant with their self-report. A prospective observational study is therefore warranted to provide more accurate results.

## Data availability statement

The raw data supporting the conclusions of this article will be made available by the authors, without undue reservation.

## Ethics statement

The studies involving humans were approved by University of Minnesota Institutional Review Board. The studies were conducted in accordance with the local legislation and institutional requirements. Participants checked a box while taking the survey that they understood that by submitting the survey their informed consent was implied.

## Author contributions

JH: Writing – original draft, Writing – review & editing. LG: Data curation, Project administration, Writing – review & editing. YA: Data curation, Writing – review & editing. MS: Conceptualization, Methodology, Writing – review & editing. JM: Conceptualization, Data curation, Investigation, Methodology, Project administration, Writing – original draft, Writing – review & editing.
